# Developing an explainable machine learning model using body composition to predict cardiovascular mortality in initial dialysis patients: a multicenter study

**DOI:** 10.3389/fphys.2026.1769240

**Published:** 2026-02-18

**Authors:** Xiao-xu Wang, Jin-xuan Wei, Tian-ke Yu, Guo-hao Zheng, Jing-yuan Cao, Min Li, Yao Wang, Shi-mei Hou, Jian Xu, Xiang-dong Yang, Bin Wang

**Affiliations:** 1 Department of Nephrology, Qilu Hospital of Shandong University, Shandong University, Jinan, China; 2 Department of Nephrology, Zhongda Hospital, School of Medicine, Southeast University, Nanjing, China; 3 Department of Nephrology, The Affiliated Taizhou People’s Hospital of Nanjing Medical University, Taizhou School of Clinical Medicine, Nanjing Medical University, Taizhou, China; 4 Department of Nephrology, The Third Affiliated Hospital of Soochow University, Soochow University, Changzhou, China; 5 Department of Nephrology, The First People’s Hospital of Changzhou, Changzhou, China; 6 Department of Nephrology, The Affiliated Hospital of Yangzhou University, Yangzhou University, Yangzhou, China; 7 Department of intensive care unit, Geriatric Hospital of Nanjing Medical University, Nanjing, China

**Keywords:** cardiovascular disease mortality, dialysis, machine learning, risk prediction, skeletal muscle density

## Abstract

**Introduction:**

Cardiovascular disease (CVD) is the leading cause of death in patients receiving dialysis, and accurate risk prediction at dialysis initiation remains limited. We developed and validated a machine learning model integrating CT-derived body composition features to predict CVD-related mortality in initial dialysis patients.

**Methods:**

Patients initiating dialysis between 2014 and 2020 from three tertiary hospitals were used for model training and internal validation, with patients from a fourth center for external validation. Clinical characteristics and laboratory variables were collected, and body composition parameters were assessed using opportunistic CT scans. Feature selection was performed using univariable logistic regression and LASSO regression. Eight machine learning algorithms were trained, and model performance was assessed using discrimination, calibration, and decision curve analysis. Model interpretability was evaluated using Shapley Additive Explanations (SHAP), and a web-based risk calculator was developed.

**Results:**

Among 1051 incident dialysis patients, 645 were assigned to the training and internal validation cohorts and 406 to the external validation cohort. Eight key predictors were identified, including age, diabetes, CVD, history of cardiac intervention, dialysis modality, skeletal muscle density, hemoglobin, and serum creatinine. CatBoost demonstrated the best performance, with an area under the receiver operating characteristic curve of 0.843 in internal validation and 0.799 in external validation, along with good calibration and clinical net benefit. SHAP analysis identified CVD, skeletal muscle density, and hemoglobin as major contributors.

**Discussion:**

An explainable machine learning model incorporating CT-derived body composition features accurately predicts CVD-related mortality in initial dialysis patients. This model may facilitate early risk stratification and targeted prevention strategies at dialysis initiation.

## Introduction

End-stage renal disease (ESRD) represents a significant global public health challenge, with its prevalence and disease burden exhibiting a persistent upward trend. Statistics indicate that over 3 million ESRD patients worldwide currently depend on maintenance dialysis therapy to sustain life (GBD Chronic Kidney Disease Collaboration, 2020; Liyanage et al., 2015; Jha et al., 2013). The dialysis population frequently suffers from multi-system dysfunction, with cardiovascular complications being particularly prevalent (Jankowski et al., 2021). Studies report that the incidence of cardiovascular death in dialysis patients is 10–20 times higher compared to the general population (Cheung et al., 2004). Cardiovascular disease (CVD) has become the leading cause of mortality in dialysis patients, accounting for more than half of all deaths (Jankowski et al., 2021; Lai et al., 2021). Therefore, early identification and accurate prediction of cardiovascular mortality risk in dialysis patients are of great importance.

Several studies have developed prediction models for cardiovascular mortality in dialysis patients. Yu et al. developed a prediction model for the 2-year risk of cardiovascular death in incident peritoneal dialysis patients, and the model had a C statistic greater than 0.70 in an independent validation cohort (Yu et al., 2018). Another study developed a cardiovascular mortality nomogram for hemodialysis patients, and the model had area under the receiver operating characteristic curve (AUC) values of 0.702, 0.695 and 0.677 for the 3-year, 5-year and 8-year predictions (Yang et al., 2023). Li et al. constructed a prediction model for the 5-year, 7-year and 9-year risk of cardiovascular death in patients with chronic kidney disease (CKD), and the external validation reported AUC values of 0.76, 0.73 and 0.73 (Li et al., 2022). However, existing models mainly rely on conventional clinical indicators and their predictive performance remains modest. Furthermore, no studies have yet incorporated patient body composition characteristics as predictive factors. With advancements in imaging technology, body composition parameters such as CT-assessed skeletal muscle index (SMI), skeletal muscle density (SMD), subcutaneous adipose tissue, and visceral adipose tissue are gaining attention as potential predictors, holding promise for enhancing the identification of cardiovascular mortality risk. Our previous multicenter study showed that SMD assessed at the first lumbar vertebra (L1) level on chest CT was independently associated with the risk of cardiac death in initial dialysis patients (Sheng et al., 2023), indicating its potential value as an imaging marker of muscle quality.

Moreover, the advent of machine learning has enabled in-depth mining of clinical data, allowing for accurate prediction of complex disease outcomes based on larger-scale, higher-dimensional datasets. Traditional prediction models typically rely on predefined hypotheses, selecting a limited number of variables and deducing their mathematical relationship with a specific outcome. In contrast, machine learning does not depend on prior assumptions but rather emphasizes learning from trends and associations within the data (Bzdok et al., 2018). By acquiring data containing outcomes, machine learning can “learn” implicit and non-linear relationships between data and outcomes, thereby enabling risk prediction for unknown samples (Gautam et al., 2022). In recent years, machine learning technology has gained increasing prominence in the medical field, being extensively used to construct various prediction models and demonstrating significant advantages, particularly in disease prognosis assessment (Liesle et al., 2024; Lieslehto et al., 2025; Huang et al., 2024). Machine learning can aid in identifying key prognostic factors, enhancing the accuracy of individualized risk stratification, thereby supporting the development of more targeted clinical intervention strategies. In this study, we integrated clinical data and CT-derived body composition measures from initial dialysis patients to build a cardiovascular mortality prediction model using machine learning methods.

## Methods

### Study cohort

This retrospective multicenter cohort study included patients who initiated dialysis between January 2014 and December 2020 in the nephrology and hemodialysis departments of four clinical centers in China.

Inclusion criteria included: (1) patients aged 18–75 years who newly started maintenance dialysis, and (2) patients who underwent a non-contrast chest or abdominal multidetector CT scan that included the L1 level within 1 month of dialysis initiation. Exclusion criteria were: (1) acute infection during the peridialytic period, (2) malignant tumors, (3) hepatic failure, (4) conditions that impair intestinal nutrient absorption such as inflammatory bowel disease, chronic diarrhea, or short bowel syndrome, (5) kidney transplantation or withdrawal from dialysis, (6) a change in dialysis modality, and (7) lack of valid follow-up records.

**FIGURE 1:**
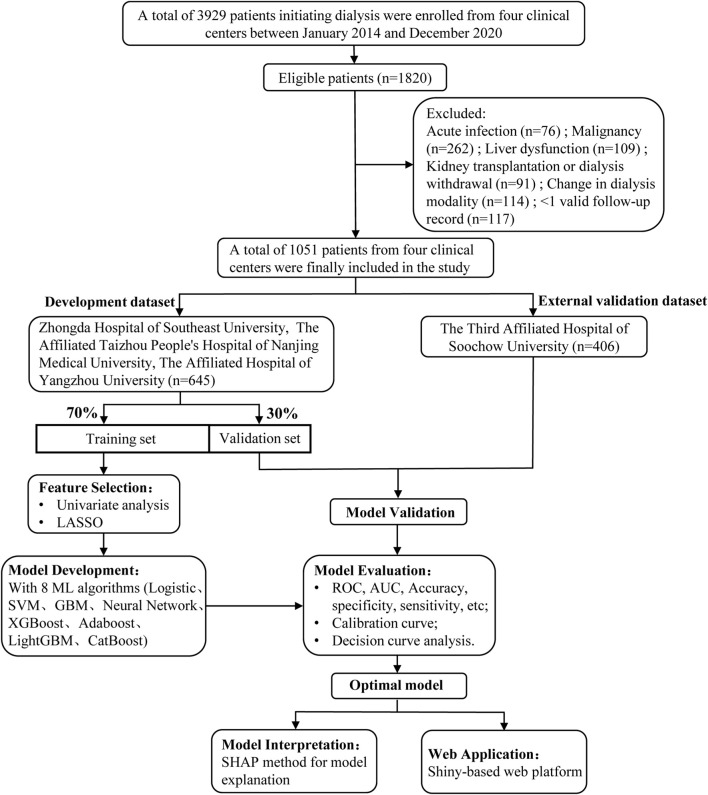
Flowchart of the study participants. SVM indicates support vector machine; GBM, Gradient Boosting Machine; XGBoost, extreme gradient boosting; Adaboost, Adaptive Boosting; CatBoost, Categorical Boosting.

As shown in Figure 1, a total of 3929 initial dialysis patients were screened from the nephrology and dialysis units of four tertiary hospitals in China. Among them, 1820 patients met the inclusion criteria. After applying the exclusion criteria, 1051 patients were finally included in this study.

This study was approved by the Ethics Committee of Zhongda Hospital (approval number 2022ZDSYLL003-P01) and was registered in the Chinese Clinical Trial Registry (registration number ChiCTR2300068453). The study complied with the principles of the Declaration of Helsinki. Because of the retrospective study design, the ethics committee waived the requirement for written informed consent.

### Clinical baseline data

All study data were collected by trained research personnel. Data collection procedures and equipment were standardized across the four study sites. Demographic and clinical information at enrollment was obtained from patients’ medical records, including age, sex, height, weight, smoking history, alcohol use, dialysis modality (hemodialysis or peritoneal dialysis), history of diabetes, hypertension, coronary artery disease, chronic heart failure, stroke, cardiac intervention, CVD, hyperlipidemia, and anemia, as well as the use of beta blockers, angiotensin converting enzyme inhibitors or angiotensin receptor blockers, calcium channel blockers, diuretics, erythropoietin, iron supplements, antiplatelet agents, Compound α-keto acid, and glucocorticoids. Body mass index (BMI) was calculated as weight in kilograms divided by height in meters squared.

White blood cell count (WBC), hemoglobin (Hb), platelet count, albumin (ALB), fasting plasma glucose (FPG), uric acid (UA), triglycerides (TG), total cholesterol (TC), low density lipoprotein cholesterol (LDL-C), serum creatinine (SCr), cystatin C (CysC), the ratio of serum creatinine to cystatin C (SCr/CysC), and blood urea nitrogen were measured within 1 week before the initiation of dialysis using standard laboratory methods.

### CT-based quantification of body composition parameters

CT examinations were performed using Discovery CT750, Revolution CT, and Optima CT 660 scanners from GE Healthcare in Milwaukee, Wisconsin, the SOMATOM Sensation scanner from Siemens Healthineers in Erlangen, Germany, or the Ingenuity CT system from Philips in Amsterdam, the Netherlands. All scans were acquired with standard settings, including 120 kVp, automatic dose modulation systems (automA and smartmA for GE Healthcare scanners, CareDose 4D for Siemens Healthineers scanners, and DoseRight for Philips scanners), a 512 × 512 matrix, a collimation of 0.625 mm, and a slice thickness of 5 mm. CT images were imported into ImageJ software (version 1.46, developed by the National Institutes of Health). The L1 level was first identified, and the axial slice showing the largest transverse diameter of the L1 transverse processes was selected for analysis. Tissue areas were then identified and quantified according to preset Hounsfield Unit ranges. The radiodensity of each tissue type was expressed as the mean CT attenuation within its corresponding HU range. The HU thresholds used to classify different tissues followed commonly accepted values in published studies. Skeletal muscle was defined as −29 to +150 HU (Golder et al., 2022). Low attenuation muscle was defined as −29 to +29 HU (Kim et al., 2020). Subcutaneous fat was identified using a threshold of −190 to −30 HU, visceral fat using −150 to −50 HU (Weinberg et al., 2018), and total fat tissue using −190 to −30 HU (Crawford et al., 2020).

### Assessment of study outcomes

The primary endpoint of this study was CVD death. CVD death was defined as death caused by acute coronary syndrome, sudden cardiac death, life-threatening arrhythmias, congestive heart failure, or ischemic/hemorrhagic stroke (Tsai et al., 2020; Sato et al., 2016). Sudden cardiac death was defined as cardiac arrest occurring within 1 hour after the onset of symptoms (Ramesh et al., 2016). Life-threatening arrhythmias were defined as documented ventricular tachycardia or ventricular fibrillation. Congestive heart failure was confirmed by electrocardiography, chest radiography, or echocardiography together with symptoms such as dyspnea or edema. Stroke was diagnosed based on characteristic imaging findings and clinical examination. All enrolled patients were followed from the date of dialysis initiation. Follow up ended at the earliest occurrence of the study endpoint, death, withdrawal from the study, loss to follow up, or the end of the follow up period on 31 December 2022. To ensure consistency across centers, a standardized definition of CVD death and a unified data extraction protocol were applied at all participating sites. This research adhered to the Transparent Reporting of a Multivariable Prediction Model for Individual Prognosis or Diagnosis (TRIPOD) Statement (Moons et al., 2015).

### Selection of variables

In the training set, univariate logistic regression was first used to identify variables that might be considered for model development. Variables with a *P* value less than 0.05 in the univariate analysis for CVD death were regarded as significant and entered the subsequent selection process. To reduce overfitting and improve predictive performance, the Least Absolute Shrinkage and Selection Operator (LASSO) method was then applied for further variable selection (Tibshirani, 1996). This method applies a penalty to the regression coefficients, which helps address multicollinearity among variables and improves model simplicity by automatically removing predictors with weak statistical contribution or high collinearity (He et al., 2023). In addition, multicollinearity among the selected variables was assessed using the variance inflation factor, and a value less than 2 indicated no evident multicollinearity.

### Development and validation of the prediction model

In this study, eight machine learning models were used to predict the risk of cardiovascular mortality in patients starting dialysis. These models included Support Vector Machine (SVM), Gradient Boosting Machine (GBM), Neural Network, Extreme Gradient Boosting (XGBoost), Adaptive Boosting (AdaBoost), Light Gradient Boosting Machine (LightGBM), Categorical Boosting (CatBoost), and Logistic Regression. In this study, the number of patients who experienced cardiovascular death was much smaller than the number who did not, resulting in an imbalance between the positive and negative samples that could affect the accuracy of the prediction models. To address this issue, the Synthetic Minority Over sampling Technique was used to balance the dataset (Chawla et al., 2002).

### Evaluation of the model

Model performance was evaluated in both the internal and external validation sets. Discrimination was assessed using the AUC. Classification performance was evaluated using accuracy, precision, sensitivity, specificity, and the F1 score. Calibration was examined with calibration curves, and clinical usefulness at different threshold probabilities was assessed with decision curve analysis. These complementary measures were used to provide a comprehensive evaluation of the predictive ability and clinical applicability of each model. Based on performance across these metrics in the training and testing sets, we selected the model with the best overall predictive performance.

### Interpretation of the model

Model interpretability was evaluated using the Shapley Additive Explanations (SHAP) method. SHAP is a model independent approach based on game theory that can be used to assess the overall importance of features and to explain predictions for individual samples. This method helps reduce the black box nature of machine learning models, improves transparency, and provides insight into how each input variable contributes to the predicted outcome. It also allows a deeper understanding of the model’s decision process and the interactions among features (Lundberg et al., 2018).

### Development of the web calculator

To enhance the clinical applicability of the prediction model, we developed an online calculator based on the final model. This web-based tool allows users to enter relevant clinical information and obtain real time estimates of the predicted probability of the outcome. The calculator was implemented using the Shiny framework in R, with the user interface built through the ui.R file and the server logic handled by the server.R file. The trained model object was loaded to generate predictions. The final application was deployed on the shinyapps.io platform and can be accessed from any device with an internet connection.

### Statistics

The extent of missing data for each variable in the original dataset is summarized in Supplementary Table S1. Variables with missing rates exceeding 25% were excluded, and the remaining missing values were handled using multiple imputation. Normally distributed continuous variables were summarized as means with standard deviations, whereas skewed continuous variables were presented as medians with interquartile ranges (IQR). Categorical variables were reported as frequencies and percentages. Between group comparisons for continuous variables were performed using the independent samples t-test or the Mann-Whitney U test, depending on distribution. Categorical variables were compared using the chi-square test. Given the limitations of *P* values in detecting differences between groups in large sample studies, the standardized mean difference was included as an additional measure to evaluate the magnitude of group differences. As a standardized effect size, the standardized mean difference is not influenced by sample size, measurement scale, or variance, and therefore provides a more objective assessment of group imbalance. An absolute standardized mean difference below 0.20 was considered small, and a value below 0.10 indicated that the difference was negligible (Cohen, 1988). All statistical analyses were performed using R software version 4.3.1 (R Foundation for Statistical Computing, Vienna, Austria) or STATA version 16.0 (StataCorp LLC, College Station, Texas, United States). A two-sided *P* value less than 0.05 was regarded as statistically significant.

## Results

### Baseline clinical characteristics

The training and internal validation cohorts consisted of 645 patients, including 452 patients in the training set and 193 patients in the internal validation set. The median age was 55 years (IQR, 45–65). Overall, the median follow-up time was 44.6 months (IQR, 29.2–63.0). During follow up, 94 patients (14.6%) experienced CVD death. Compared with patients who did not experience CVD death, those who did were older and had lower levels of Hb, SCr, SCr/CysC, and SMD. They also had higher levels of low attenuation muscle area, the low attenuation muscle to skeletal muscle area ratio, subcutaneous fat area, and total fat area (Table 1; Supplementary Figure S1). In addition, the prevalence of diabetes, coronary artery disease, chronic heart failure, and CVD was higher in patients who experienced CVD death, as were the proportions of those with a history of cardiac intervention and those using antiplatelet agents (Table 1; Supplementary Figure S2).

**TABLE 1:** Characteristics of participants in the development dataset.

Characteristic	Total (N = 645)	CVD death (N = 94)	Non-CVD death (N = 551)	*P* value	SMD (Std.)^a^
Age, years	55.0 (45.0–65.0)	63.5 (56.0–69.0)	54.0 (44.0–63.5)	<0.001	−0.604
Sex, n (%)				0.557	0.066
Male	395 (61.2%)	55 (58.5%)	340 (61.7%)		
Female	250 (38.8%)	39 (41.5%)	211 (38.3%)		
BMI, kg/m^2^	23.8 (21.3–26.5)	23.9 (21.1–27.0)	23.7 (21.3–26.4)	0.649	−0.005
Smoking history, n (%)	115 (10.9%)	21 (22.3%)	94 (17.1%)	0.216	−0.138
Alcohol history, n (%)	48 (4.6%)	11 (11.7%)	37 (6.7%)	0.089	−0.190
Dialysis modality, n (%)				0.002	−0.345
Hemodialysis	571 (88.5%)	92 (97.9%)	479 (86.9%)		
Peritoneal dialysis	74 (11.5%)	2 (2.1%)	72 (13.1%)		
β-blockers, n (%)	398 (37.9%)	62 (66.0%)	336 (61.0%)	0.359	−0.102
ACEI/ARB, n (%)	218 (20.7%)	35 (37.2%)	183 (33.2%)	0.446	−0.085
CCB, n (%)	556 (52.9%)	81 (86.2%)	475 (86.2%)	0.992	0.001
Diuretics, n (%)	262 (24.9%)	43 (45.7%)	219 (39.7%)	0.274	−0.122
EPO, n (%)	549 (52.2%)	80 (85.1%)	469 (85.1%)	0.998	0.000
Iron agent, n (%)	368 (35.0%)	55 (58.5%)	313 (56.8%)	0.758	−0.034
Antiplatelet agents, n (%)	167 (15.9%)	36 (38.3%)	131 (23.8%)	0.003	−0.333
Compound α-keto acid, n (%)	273 (26.0%)	37 (39.4%)	236 (42.8%)	0.529	0.070
Glucocorticoids, n (%)	91 (8.7%)	13 (13.8%)	78 (14.2%)	0.933	0.009
Diabetes mellitus, n (%)	298 (28.4%)	66 (70.2%)	232 (42.1%)	<0.001	−0.574
Hypertension, n (%)	590 (56.1%)	88 (93.6%)	502 (91.1%)	0.421	−0.090
Coronary artery disease, n (%)	125 (11.9%)	32 (34.0%)	93 (16.9%)	<0.001	−0.439
Chronic heart failure, n (%)	197 (18.7%)	43 (45.7%)	154 (27.9%)	<0.001	−0.389
Stroke, n (%)	63 (6.0%)	14 (14.9%)	49 (8.9%)	0.070	−0.202
Cardiac intervention, n (%)	38 (3.6%)	12 (12.8%)	26 (4.7%)	0.002	−0.344
CVD, n (%)	297 (28.3%)	73 (77.7%)	224 (40.7%)	<0.001	−0.768
Hyperlipidemia, n (%)	37 (3.5%)	3 (3.2%)	34 (6.2%)	0.251	0.128
Anemia history, n (%)	542 (51.6%)	76 (80.9%)	466 (84.6%)	0.363	0.102
WBC,*10^9^/L	6.6 (5.3–8.3)	6.6 (5.6–8.3)	6.5 (5.3–8.3)	0.301	−0.092
Hemoglobin, g/L	83.0 (74.0–94.0)	81.0 (73.0–86.2)	84.0 (74.0–96.0)	0.002	0.451
PLT,*10^9^/L	169.0 (124.0–209.5)	169.0 (126.0–224.0)	169.0 (123.5–208.0)	0.495	−0.097
Albumin, g/L	33.2 (29.6–37.1)	32.3 (6.2)	33.5 (5.7)	0.081	0.195
FPG, mg/dl	5.4 (4.6–6.9)	5.6 (4.7–8.4)	5.3 (4.6–6.7)	0.148	−0.200
Uric acid, mmol/L	466.0 (378.4–561.5)	480.4 (372.0–582.0)	464.0 (381.0–554.0)	0.745	−0.066
Triglycerides, mmol/L	1.5 (1.1–2.0)	1.6 (1.2–1.9)	1.4 (1.0–2.0)	0.381	0.017
Total cholesterol, mmol/L	4.0 (3.3–4.9)	3.9 (3.2–5.1)	4.0 (3.3–4.9)	0.973	−0.057
LDL cholesterol, mmol/L	2.3 (1.8–3.0)	2.3 (1.8–2.9)	2.3 (1.8–3.0)	0.981	−0.049
SCr, mg/dL	9.0 (7.6–11.5)	8.0 (6.9–9.8)	9.2 (7.7–11.9)	<0.001	0.390
CysC, mg/L	4.9 (3.8–6.2)	4.4 (3.5–5.9)	5.0 (3.9–6.2)	0.073	0.115
SCr/CysC	19.6 (14.5–25.1)	19.2 (12.6–23.1)	19.7 (14.8–25.6)	0.037	0.143
BUN, mmol/L	28.8 (21.4–36.4)	26.4 (17.1–36.6)	29.2 (21.8–36.3)	0.057	0.202
SMI, cm^2^/m^2^	40.3 (34.6–45.2)	40.5 (34.9–45.1)	40.3 (34.6–45.2)	0.747	0.031
SMD, HU	34.1 ± 8.3	29.8 ± 7.6	34.8 ± 8.2	<0.001	0.617
LAMA, cm^2^	46.6 (36.6–59.3)	51.8 (40.1–63.2)	45.7 (35.9–58.3)	0.009	−0.252
LAMD, HU	6.4 (4.6–7.9)	6.2 (4.4–7.8)	6.4 (4.6–7.9)	0.703	0.108
LAMA/SMA	0.4 (0.4–0.5)	0.5 (0.4–0.6)	0.4 (0.3–0.5)	<0.001	−0.406
SFA, cm^2^	70.4 (43.7–110.8)	85.2 (48.2–129.4)	69.3 (42.0–106.9)	0.031	−0.119
SFD, HU	−84.0 (−94.5 to −73.4)	−83.0 (−96.1 to −75.0)	−84.1 (−94.0 to −72.8)	0.640	0.052
VFA, cm^2^	75.0 (33.9–135.7)	93.0 (42.3–138.4)	72.4 (33.1–133.6)	0.061	−0.217
VFD, HU	−85.5 (−93.0 to −77.4)	−86.6 (−92.2 to −77.9)	−85.5 (−93.0 to −77.2)	0.936	−0.016
TFA, cm^2^	185.9 (105.7–284.1)	212.6 (125.6–312.2)	182.7 (103.4–280.4)	0.041	−0.185
TFD, HU	−80.8 (−89.9 to −69.3)	−80.7 (−89.2 to −72.0)	−80.8 (−90.1 to −69.2)	0.861	0.008

^a^ SMD (Std.): Standardized mean difference (effect size measure). BMI, indicates body mass index; ACEI, angiotensin-converting enzyme inhibitor; ARB, angiotensin II, receptor blocker; CCB, calcium channel blocker; EPO, erythropoietin; CVD, cardiovascular disease; WBC, white blood cell count; PLT, platelet count; FPG, fasting plasma glucose; LDL, low-density lipoprotein; SCr, serum creatinine; CysC, cystatin C; BUN, blood urea nitrogen; SMI, skeletal muscle index; SMD, skeletal muscle radiodensity; LAMA, low-attenuation muscle area; LAMD, low-attenuation muscle density; SMA, skeletal muscle area; SFA, subcutaneous fat area; SFD, subcutaneous fat density; VFA, visceral fat area; VFD, visceral fat density; TFA, total fat area; TFD, total fat density.

The external validation cohort consisted of 406 patients, with a median age of 52 years (IQR, 42–63). During follow up, 40 patients (9.9%) experienced CVD death. Detailed patient characteristics are shown in Supplementary Table S2. Differences between the development dataset and the external validation dataset are summarized in Supplementary Table S3. Several variables differed significantly between the two datasets, such as age, sex, BMI, smoking and alcohol history, WBC, ALB, UA, LDL-C, CysC, SCr/CysC, SMI, SMD, low attenuation muscle area, low attenuation muscle density, the low attenuation muscle to skeletal muscle area ratio, subcutaneous fat area, subcutaneous fat density, visceral fat area, visceral fat density, total fat area, total fat density, diabetes, coronary artery disease, history of cardiac intervention, CVD, and the use of iron agents, antiplatelet agents, Compound α-keto acid, and glucocorticoids.

### Selection of important features

**FIGURE 2:**
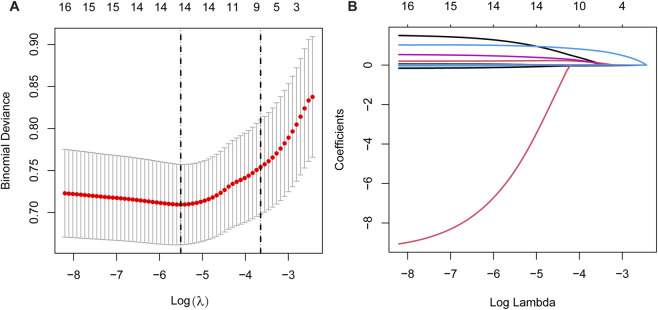
LASSO regression analysis for predictor selection. As shown in **(A)**, the plot illustrates the relationship between the binomial deviance and log(λ). The vertical dashed lines clearly mark the two λ values selected based on the minimum deviance criterion and the one-standard-error rule. This approach helps balance model accuracy while reducing the risk of overfitting. **(B)** presents the trajectories of variable coefficients across the log(λ) sequence, with each colored line representing a different predictor. As λ increases, the coefficients of weakly relevant or irrelevant predictors progressively shrink toward zero, thereby achieving feature selection and ultimately retaining only the most important variables in the model.

Using univariable logistic regression analysis, 17 variables associated with cardiovascular death in initial dialysis patients were identified, all with *P* values less than 0.05 (Table 2). LASSO regression was then applied to further select key predictors, resulting in eight variables: age, diabetes, CVD, history of cardiac intervention, dialysis modality, SMD, Hb and SCr (Figure 2). Multicollinearity was evaluated for these eight variables. Spearman correlation analysis showed correlation coefficients below 0.4, and all variables had tolerance values greater than 0.6 and variance inflation factors below 1.5, indicating no significant multicollinearity (Supplementary Figure S3; Supplementary Table S4). These eight variables were therefore used to construct the prediction model for CVD-related mortality in initial dialysis patients.

**TABLE 2:** Univariate logistic regression analysis for the training set.

Covariables	OR	95% CI	*P* value
Age, years	1.06	1.03–1.08	<0.001
Sex (male)	0.85	0.50–1.45	0.550
BMI, kg/m^2^	1.00	0.93–1.06	0.886
Smoking history	1.51	0.82–2.78	0.182
Alcohol history	1.87	0.85–4.15	0.122
Dialysis modality (hemodialysis)	5.54	1.32–23.29	0.019
β-blockers	1.53	0.87–2.68	0.141
ACEI/ARB	1.00	0.58–1.73	0.988
CCB	0.78	0.39–1.55	0.477
Diuretics	1.03	0.61–1.76	0.908
EPO	1.47	0.67–3.22	0.338
Iron agent	1.14	0.67–1.93	0.635
Antiplatelet agents	2.12	1.23–3.65	0.007
Compound α-keto acid	0.94	0.55–1.59	0.810
Glucocorticoids	1.17	0.56–2.45	0.670
Diabetes mellitus	2.65	1.53–4.59	0.001
Hypertension	1.22	0.46–3.23	0.694
Coronary artery disease	2.75	1.57–4.82	0.001
Chronic heart failure	2.04	1.20–3.47	0.009
Stroke	1.63	0.74–3.59	0.220
Cardiac intervention	3.05	1.32–7.07	0.009
CVD	4.65	2.53–8.56	0.001
Hyperlipidemia	0.75	0.22–2.58	0.650
Anemia history	0.78	0.40–1.52	0.464
WBC, *10^9^/L	1.07	0.99–1.17	0.087
Hemoglobin, g/L	0.97	0.95–0.99	0.002
PLT, *10^9^/L	1.00	1.00–1.01	0.031
Albumin, g/L	0.99	0.95–1.03	0.616
FPG, mg/dl	1.08	1.00–1.16	0.063
Uric acid, mmol/L	1.00	1.00–1.00	0.946
Triglycerides, mmol/L	1.00	0.79–1.27	0.986
Total cholesterol, mmol/L	1.00	0.83–1.21	0.979
LDL cholesterol, mmol/L	1.02	0.78–1.33	0.897
SCr, mg/dL	0.85	0.78–0.94	0.001
CysC, mg/L	0.97	0.84–1.13	0.738
SCr/CysC	0.98	0.95–1.00	0.102
BUN, mmol/L	0.98	0.95–1.00	0.046
SMI, cm^2^/m^2^	0.99	0.96–1.02	0.345
SMD, HU	0.92	0.89–0.95	0.001
LAMA, cm^2^	1.01	0.99–1.02	0.251
LAMD, HU	0.95	0.86–1.05	0.298
LAMA/SMA	10.95	1.78–67.50	0.010
SFA, cm^2^	1.00	1.00–1.01	0.036
SFD, HU	0.98	0.97–1.00	0.093
VFA, cm^2^	1.00	1.00–1.01	0.015
VFD, HU	0.98	0.96–1.01	0.159
TFA, cm^2^	1.00	1.00–1.00	0.013
TFD, HU	0.98	0.96–1.00	0.097

BMI, indicates body mass index; ACEI, angiotensin-converting enzyme inhibitor; ARB, angiotensin II, receptor blocker; CCB, calcium channel blocker; EPO, erythropoietin; CVD, cardiovascular disease; WBC, white blood cell count; PLT, platelet count; FPG, fasting plasma glucose; LDL, low-density lipoprotein; SCr, serum creatinine; CysC, cystatin C; BUN, blood urea nitrogen; SMI, skeletal muscle index; SMD, skeletal muscle radiodensity; LAMA, low-attenuation muscle area; LAMD, low-attenuation muscle density; SMA, skeletal muscle area; SFA, subcutaneous fat area; SFD, subcutaneous fat density; VFA, visceral fat area; VFD, visceral fat density; TFA, total fat area; TFD, total fat density.

### Development and validation of predictive models

**FIGURE 3:**
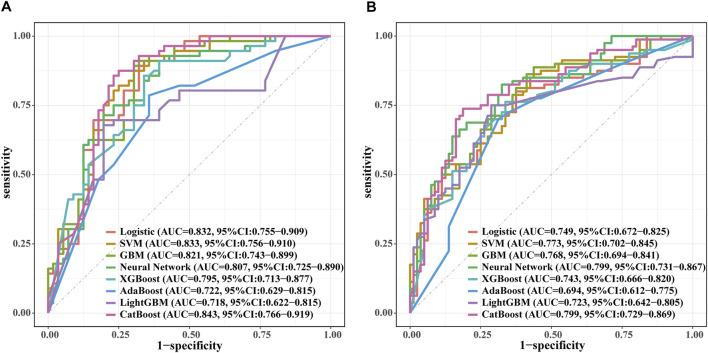
Comparison of ROC curves for eight machine learning models in the internal and external validation sets. SVM indicates support vector machine; GBM, Gradient Boosting Machine; XGBoost, extreme gradient boosting; Adaboost, Adaptive Boosting; CatBoost, Categorical Boosting. **(A)** Receiver operating characteristic curve in internal validation. **(B)** Receiver operating characteristic curve in external validation.

**FIGURE 4:**
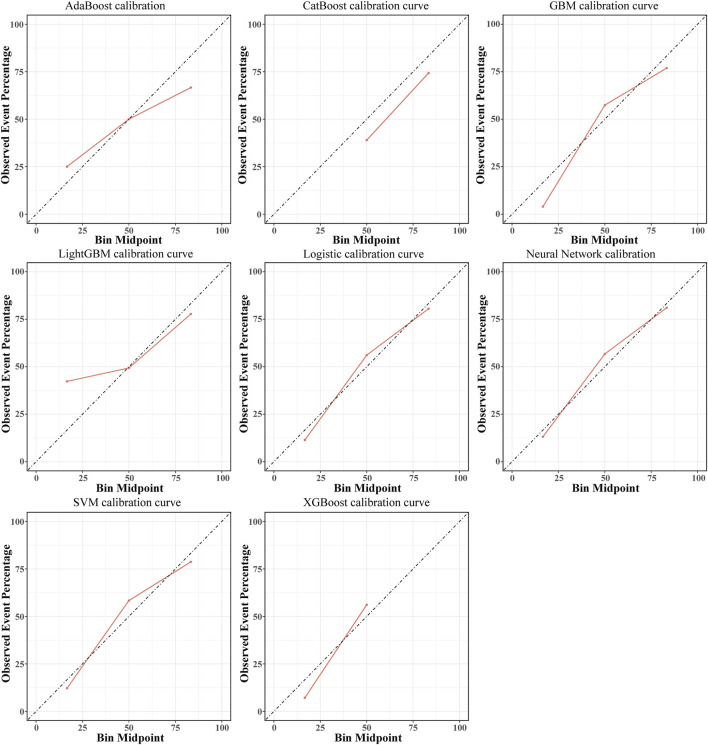
Calibration curves of eight machine learning models in the internal validation set. The horizontal axis represents the predicted probability of CVD mortality, and the vertical axis shows the observed event probability based on actual outcomes. The dashed line indicates the ideal reference line, where predicted probabilities perfectly match the observed outcomes. The closer each model’s calibration curve is to the dashed line, the better its calibration performance. SVM indicates support vector machine; GBM, Gradient Boosting Machine; XGBoost, extreme gradient boosting; Adaboost, Adaptive Boosting; CatBoost, Categorical Boosting; CVD, cardiovascular disease.

**FIGURE 5:**
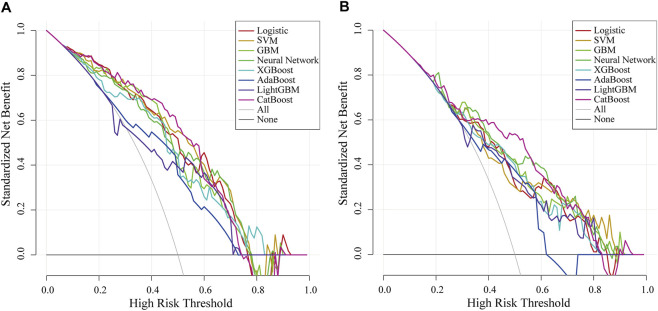
Decision curve analysis of eight machine learning models in the internal and external validation sets. The black horizontal line represents the net benefit when all patients are assumed to be non-CVD deaths, while the light gray curve represents the net benefit when all patients are assumed to be CVD deaths. The area between each model’s colored curve and the light gray curve reflects its clinical utility. A larger net benefit indicates greater clinical decision-making value of the model within the corresponding range of risk thresholds. SVM indicates support vector machine; GBM, Gradient Boosting Machine; XGBoost, extreme gradient boosting; Adaboost, Adaptive Boosting; CatBoost, Categorical Boosting; CVD, cardiovascular disease. **(A)** Decision curve analysis in internal validation. **(B)** Decision curve analysis in external validation.

After the predictive features were selected, eight machine learning models were developed, including SVM, GBM, Neural Network, XGBoost, AdaBoost, LightGBM, CatBoost, and Logistic Regression. In the internal validation set, the AUC of the eight models was compared, as shown in Figure 3A. The CatBoost model demonstrated the best discrimination, with an AUC of 0.843 (95% CI, 0.766–0.919), followed by SVM (AUC 0.833; 95% CI, 0.756–0.910) and Logistic Regression (AUC 0.832; 95% CI, 0.755–0.909). Performance metrics for the internal validation set are presented in Table 3. Among all models, CatBoost showed the most favorable overall performance, achieving the highest accuracy (0.812), precision (0.778), and F1 score (0.824), with sensitivity (0.875) and specificity (0.750) also at comparatively high levels. The calibration curves for the internal validation set are shown in Figure 4. The predicted probabilities of the CatBoost model aligned closely with the reference line, indicating good calibration and suggesting that the model captured the individual risk levels with higher accuracy. Figure 5A presents the decision curves for predicting CVD mortality in the internal validation set. Across most threshold probabilities, the CatBoost model provided greater net benefit than the other models, demonstrating stronger clinical usefulness, particularly in settings requiring individualized risk assessment.

**TABLE 3:** Comparison of performance indicators of eight machine learning prediction models across internal validation set.

Predictive models	Accuracy	Sensitivity	Specificity	Precision	F-1 score
Logistic	0.786	0.893	0.679	0.735	0.806
SVM	0.786	0.929	0.643	0.722	0.813
GBM	0.795	0.911	0.679	0.739	0.816
Neural network	0.759	0.911	0.607	0.699	0.791
XGBoost	0.759	0.911	0.607	0.699	0.791
AdaBoost	0.714	0.786	0.643	0.688	0.733
LightGBM	0.741	0.679	0.804	0.776	0.724
CatBoost	0.812	0.875	0.750	0.778	0.824

SVM, indicates support vector machine; GBM, gradient boosting machine; XGBoost, extreme gradient boosting; Adaboost, Adaptive Boosting; CatBoost, Categorical Boosting.

**FIGURE 6:**
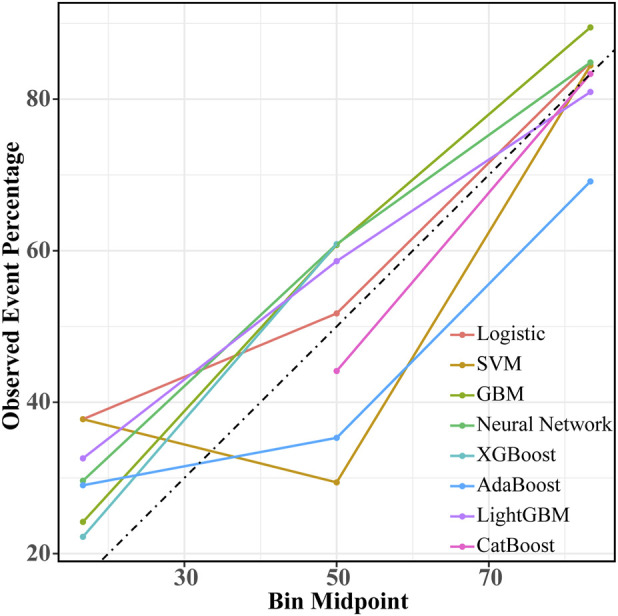
Calibration curves of eight machine learning models in the external validation set. The horizontal axis represents the predicted probability of CVD mortality, while the vertical axis shows the observed event probability based on actual outcomes. The dashed line indicates the ideal reference line, where predicted probabilities perfectly correspond to observed results. The closer each model’s calibration curve is to the dashed line, the better its calibration performance. SVM indicates support vector machine; GBM, Gradient Boosting Machine; XGBoost, extreme gradient boosting; Adaboost, Adaptive Boosting; CatBoost, Categorical Boosting; CVD, cardiovascular disease.

The ROC curves of the eight machine learning models in the external validation set are presented in Figure 3B. The CatBoost model showed the best discrimination with an AUC of 0.799 (95% CI, 0.729–0.869). The Neural Network model demonstrated comparable performance, with an AUC of 0.799 (95% CI, 0.731–0.867). Table 4 summarizes the predictive performance of each model in the external validation set. The CatBoost model achieved the highest accuracy (0.775), specificity (0.812), precision (0.797), and F1 score (0.766), suggesting strong generalizability and stable predictive performance on external data. The Neural Network model showed better sensitivity (0.825), although its accuracy (0.750), specificity (0.675), precision (0.717), and F1 score (0.767) were lower than those of the CatBoost model. Calibration curves for the external validation set are presented in Figure 6, showing that the CatBoost model exhibited calibration closer to the ideal diagonal line. Decision curve analysis for all models is shown in Figure 5B, and across most threshold probabilities, CatBoost provided a higher standardized net benefit than the other models. Given its superior performance over other models and consistent results in both validation sets, CatBoost was selected as the optimal model for interpretability analysis.

**TABLE 4:** Comparison of performance indicators of eight machine learning prediction models across external validation set.

Predictive models	Accuracy	Sensitivity	Specificity	Precision	F-1 score
Logistic	0.713	0.800	0.625	0.681	0.736
SVM	0.713	0.863	0.562	0.663	0.750
GBM	0.725	0.863	0.588	0.676	0.758
Neural network	0.750	0.825	0.675	0.717	0.767
XGBoost	0.713	0.762	0.662	0.693	0.726
AdaBoost	0.694	0.700	0.688	0.691	0.696
LightGBM	0.731	0.750	0.713	0.723	0.736
CatBoost	0.775	0.738	0.812	0.797	0.766

SVM, indicates support vector machine; GBM, gradient boosting machine; XGBoost, extreme gradient boosting; Adaboost, Adaptive Boosting; CatBoost, Categorical Boosting.

**FIGURE 7:**
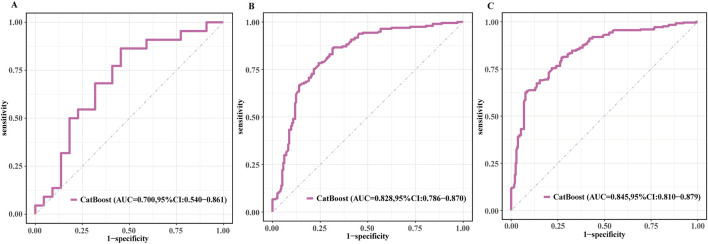
ROC curves of the CatBoost model for 1-, 3-, and 5-year CVD mortality in the external validation set. **(A)** 1-year ROC curve. **(B)** 3-year ROC curve. **(C)** 5-year ROC curve.

To further clarify predictive performance across follow-up durations, we evaluated the discrimination of the CatBoost model for CVD mortality at 1, 3, and 5 years in the external validation cohort. As shown in Figure 7, the AUC was 0.700 (95% CI, 0.540–0.861) at 1 year, 0.828 (95% CI, 0.786–0.870) at 3 years, and 0.845 (95% CI, 0.810–0.879) at 5 years. The 1-year estimate had a wider confidence interval, possibly due to fewer events in the first year, while discrimination was more stable at 3 and 5 years.

### Interpretability analysis of the CatBoost model

**FIGURE 8:**
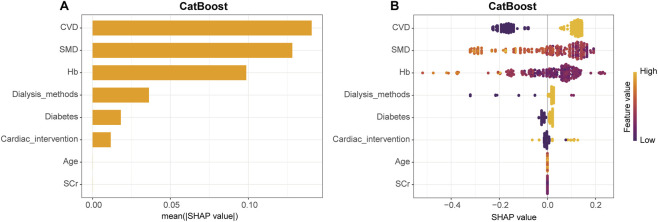
Global SHAP value explanation of predictive features. **(A)** The bar plot shows the mean SHAP values of each predictor, ranked in descending order of their contribution to the model. This reflects the relative importance of each variable in the model’s decision-making process. **(B)** The SHAP scatter plot presents the SHAP values (x-axis) for each patient in the training set. Each point represents an individual patient, with colors indicating the magnitude of the corresponding feature value (yellow for high values and purple for low values). A positive SHAP value indicates a positive effect of the feature on the predicted outcome, whereas a negative SHAP value indicates a negative effect.

We applied the SHAP approach to explain the output of the final model by quantifying the contribution of each variable. In the global explanation, the SHAP summary bar plot (Figure 8A) showed that CVD, SMD, and Hb were the three most influential predictors, indicating their substantial contribution to the estimated risk of CVD mortality. In addition, variables such as dialysis modality, diabetes, and a history of cardiac interventions also contributed to model decisions. In Figure 8B, each point represents an individual patient, with its position on the horizontal axis indicating the SHAP value for that variable and the color reflecting the magnitude of the variable (yellow for higher values and purple for lower values). For example, CVD showed a clear positive influence on adverse outcomes, meaning that patients with CVD were more likely to be classified by the model as having a higher risk of CVD mortality. The importance and direction of effect of each variable are visualized through its ranking and SHAP value distribution. Similarly, SMD and Hb had strong effects on the predictions but in the opposite direction. Lower levels of SMD or Hb were generally associated with positive SHAP values, suggesting that reduced muscle density and lower Hb levels may be important contributors to an increased risk of CVD mortality. This SHAP scatter plot illustrates how variable levels relate to predicted risk and enhances the interpretability of the model by providing a visual link between clinical features and prediction outcomes. A SHAP value heatmap (Supplementary Figure S4) was used to visualize individual-level contributions of each predictor across the cohort, with patients ordered by the model-predicted outcome. Each column represents a patient and each row a variable, with color denoting SHAP values. Patients predicted to experience CVD mortality (orange section) exhibited stronger positive SHAP values (yellow tones) for variables such as CVD, SMD, and Hb. This pattern further supports the stable and consistent contribution of these predictors to the model’s predictions.

The relationship between Hb levels and their corresponding SHAP values is shown in Supplementary Figure S5A, with SMD encoded by color to further illustrate its influence on the model output. Low Hb levels were associated with positive SHAP values, with a more pronounced effect observed among patients with lower SMD. To explore this interaction pattern in greater detail, Supplementary Figure S5B plots SMD on the horizontal axis with Hb represented by color. A similar trend was observed: patients with lower SMD and lower Hb were more likely to be classified as high risk by the model. These findings highlight a potential synergistic effect of SMD and Hb in distinguishing patients at higher risk of CVD mortality.

**FIGURE 9:**
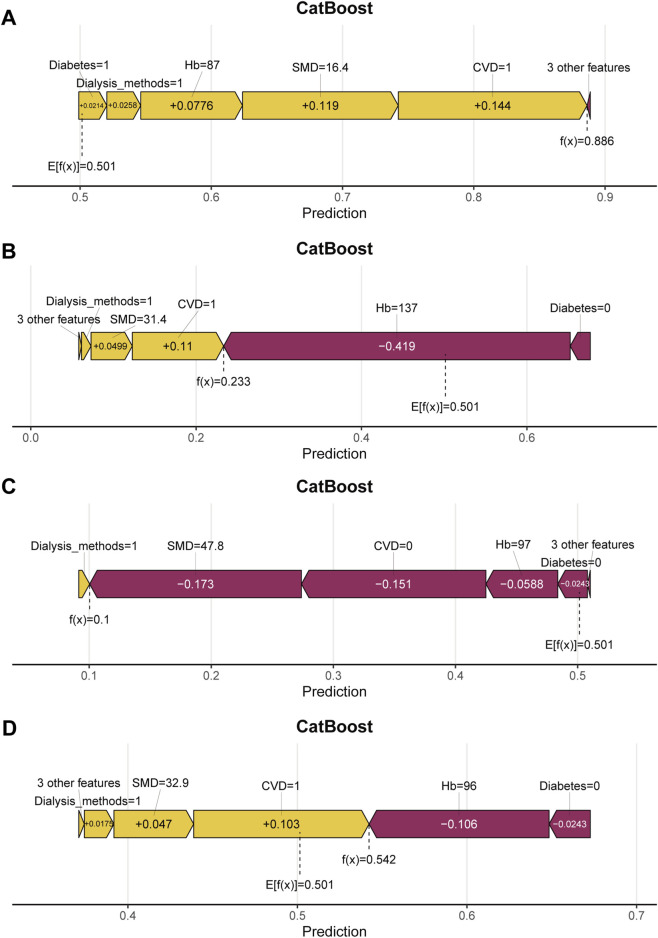
Local interpretability: SHAP force plot illustrating individual prediction explanations. **(A–D)** present four representative patients from the testing set. Yellow bars indicate features that increase the predicted risk, whereas purple bars indicate features that decrease the predicted risk. E[f(x)] is the baseline value and f(x) is the final prediction.

Figure 9 presents the SHAP force plots for four patients in the testing set, illustrating how the CatBoost model generated individualized predictions by showing the specific contribution of each variable. In these plots, yellow bars represent features that pushed the prediction toward higher risk, whereas purple bars indicate features that pushed the prediction toward lower risk. The length of each bar reflects the magnitude of the feature’s effect on the model output. The value f(x) denotes the predicted risk for that patient, and E [f(x)] represents the model’s baseline value. As shown in Figure 9A, the patient’s predicted risk was primarily driven by several risk factors with positive contributions, including a history of CVD (CVD = 1, contribution +0.144), low SMD (16.4, contribution +0.119), low Hb (87, contribution +0.0776), and hemodialysis modality (dialysis modality = 1, contribution +0.0258). Although a few variables had minor negative contributions, they were insufficient to offset the cumulative influence of these risk factors. The final model output for this patient was f(x) = 0.886, which exceeded the baseline value E [f(x)] = 0.501, indicating a high predicted probability of CVD mortality. In contrast, the force plot for another patient shown in Figure 9C indicates that several variables contributed negatively to the model output, including higher SMD (47.8, contribution −0.173) and the absence of CVD (CVD = 0, contribution −0.151). The combined influence of these protective factors substantially lowered the predicted risk. The final model output for this patient was f(x) = 0.100, which was well below the baseline value and suggested a low probability of CVD mortality. Figures 9B,D similarly demonstrate that key variables such as CVD, Hb, and SMD consistently shaped predictions across different patients, showing good discriminative ability and stable interpretability and supporting the reliability of the model for individual level risk assessment.

**FIGURE 10:**
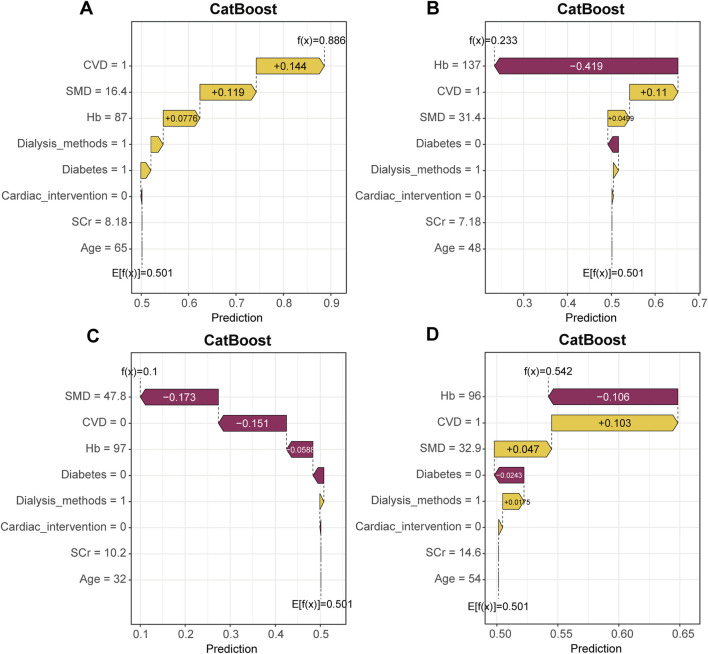
Local interpretability: SHAP waterfall plot illustrating individual prediction explanations. **(A–D)** correspond to four different patients and show how variables move the prediction from the baseline E[f(x)] to the final output f(x). Rightward shifts indicate positive contributions, and leftward shifts indicate negative contributions.

Figure 10 shows the SHAP waterfall plots for the corresponding individuals, providing a clearer view of the order and direction of each variable’s cumulative contribution to the prediction. The variables are arranged according to their contribution, beginning at the model’s baseline value E [f(x)] and moving to the right for positive contributions and to the left for negative contributions, resulting in the final prediction f(x). For example, in Figure 10A, the presence of CVD, low SMD, and low Hb markedly increased the predicted risk. In Figure 10B, higher Hb and the absence of diabetes reduced the predicted value f(x), thereby lowering the estimated risk. Similar contribution patterns were observed in Figures 10C,D, indicating that the model maintained consistent interpretability at the individual level.

### Web-based application of the predictive model

**FIGURE 11:**
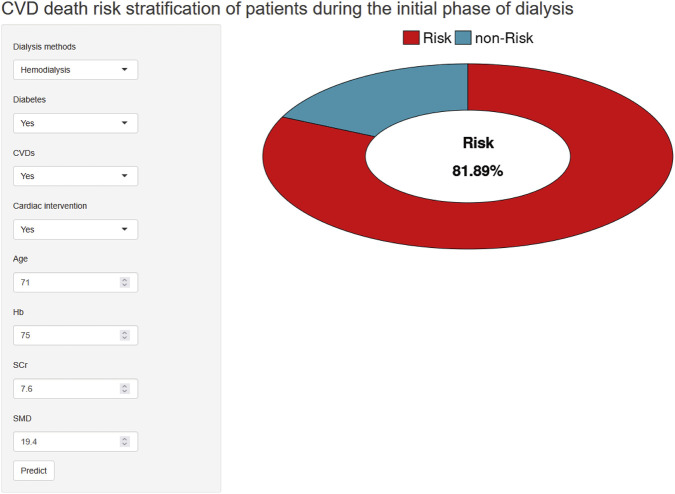
Web-based visualization interface of the CatBoost model.

As shown in Figure 11, the final predictive model was incorporated into a web-based application to facilitate use in clinical practice. Users can enter the actual values of the eight required features, and the system automatically computes the predicted risk of CVD mortality for initial dialysis patients. The online predictive tool is accessible at the following link: https://wangxiaoxu0817.shinyapps.io/workrun15/.

## Discussion

This study constructed eight machine learning models to predict CVD mortality risk in initial dialysis patients, among which the CatBoost model demonstrated the highest clinical predictive value. This model uses eight variables, including age, Hb, SCr, SMD, history of CVD, history of diabetes, history of cardiac intervention and dialysis modality, and showed an AUC of 0.843 (95% CI: 0.766–0.919) in the internal validation set and 0.799 (95% CI: 0.729–0.869) in the external validation set. Based on this, we established a clinically applicable prediction model for CVD mortality in initial dialysis patients and developed a web-based calculator tool to enhance its practicality and potential for clinical dissemination.

CKD patients constitute a high-risk population for CVD incidence and mortality (Jankowski et al., 2021), and CKD has been added to the traditional risk factors for CVD by the American College of Cardiology (Mende, 2020). CVD risk increases early in the course of CKD and escalates further with declining estimated glomerular filtration rate (eGFR) (Yaqoob et al., 2025). A prospective observational study involving approximately 1.12 million community-dwelling adults without dialysis or kidney transplantation (median follow-up 2.84 years) showed that reduced eGFR levels were independent risk factors for hospitalization due to coronary heart disease, heart failure, stroke, or peripheral artery disease. For instance, compared to individuals with eGFR ≥60 mL/min/1.73 m^2^, when eGFR was <15 mL/min/1.73 m^2^, the all-cause mortality risk increased to 5.9 times, cardiovascular event risk to 3.4 times, and hospitalization risk to 3.1 times (Go et al., 2004). When the disease progresses to the ESRD stage requiring dialysis therapy, cardiovascular event-related deaths account for over 50% of total patient mortality (Jankowski et al., 2021; Lai et al., 2021; Sha et al., 2017). It is reported that CVD-related mortality in hemodialysis patients is 20 times that of the general population (Cozzolino et al., 2018). Although the prevalence and fatality rate of CVD are extremely high in dialysis patients, they are often underestimated in clinical practice, leading to a failure to promptly identify and intervene in some high-risk patients. Previous studies have reported several all-cause mortality prediction models for dialysis patients (Wagner et al., 2011; Floege et al., 2015; Zhao et al., 2014); however, the development of risk assessment tools specifically for cardiovascular mortality in initial dialysis patients is limited. Prediction models for cardiovascular death are more helpful for etiology-specific intervention. Therefore, there is an urgent need to introduce effective risk prediction tools to strengthen early identification and intervention of CVD mortality risk, optimize resource allocation and treatment strategies, thereby improving cardiovascular outcomes in initial dialysis patients.

Based on machine learning methods, this study constructed a prediction model for CVD mortality risk in initial dialysis patients, ultimately identifying eight key predictors: age, Hb, SCr, SMD, history of CVD, history of diabetes, history of cardiac intervention, and dialysis modality. Multiple studies have identified age as a significant predictor of CVD mortality (Yu et al., 2018; Li et al., 2022; Anker et al., 2016; Xia et al., 2017). In both hemodialysis and peritoneal dialysis patients, CVD mortality increases significantly with age. Aging leads to elevated oxidative stress, cellular senescence, and impaired synthesis and secretion of vasoactive substances, gradually damaging vascular structure and function, causing hemodynamic disturbances, and thereby significantly increasing the risk of cardiovascular events in dialysis patients (Yang et al., 2023). Previous research indicates that patients undergoing renal replacement therapy who experience a CVD event have a significantly reduced 4-year survival probability of only 4%, suggesting an extremely poor prognosis (Grams et al., 2018). In this study, a history of CVD was selected as a predictor of CVD death and exhibited high variable importance in the model, being considered one of the primary predictors. Additionally, a history of cardiac intervention, reflecting prior severe cardiac disease burden, was also identified as a relevant predictor. History of diabetes, a traditional CVD risk factor, has been included in CVD mortality prediction models for dialysis patients in multiple studies (Yang et al., 2023; Anker et al., 2016; Xia et al., 2017). The ADVANCE study, which included 11,140 patients with type 2 diabetes, showed that intensive glucose control compared to standard treatment reduced the risk of a composite outcome of major macrovascular and microvascular events (Patel et al., 2008). This study further validates the significant predictive value of diabetes for CVD mortality in dialysis patients. Anemia is one of the non-traditional cardiovascular risk markers (Cozzolino et al., 2018), with some scholars proposing the concept of the “cardio-renal anemia syndrome” to emphasize the key role of anemia in the cardio-renal axis (Yogasundaram et al., 2019). Our results also show that lower Hb levels are closely associated with an increased risk of CVD death. This study found that patients who experienced CVD death had lower SCr levels compared to those who did not, which may reflect decreased muscle mass and insufficient nutritional reserves. In dialysis patients, while SCr is a uremic toxin, its level can also reflect muscle mass, nutritional status, and physical activity capacity (Patel et al., 2013; Canaud et al., 2020). Multiple studies have confirmed that lower SCr levels are associated with adverse outcomes such as higher CVD mortality or all-cause mortality (Canaud et al., 2020; Kalantar-Zadeh et al., 2003). The incidence of CVD death differs among patients on different dialysis modalities; peritoneal dialysis patients generally have a lower risk of cardiovascular death in the early dialysis period compared to hemodialysis patients. This difference may be related to factors such as volume status management, preservation of residual renal function, inflammation levels, and toxin clearance modalities (Sukul et al., 2020; Moist et al., 2000). Therefore, including dialysis modality helps improve the model’s ability to discriminate CVD mortality risk among different dialysis patients.

This study is the first to incorporate SMD into a CVD mortality risk prediction model. As an indicator reflecting skeletal muscle quality, decreased SMD may suggest muscle fat infiltration and declining motor function (Cruz-Jentoft et al., 2019). According to recent guidelines from the European Working Group on Sarcopenia in Older People (EWGSOP) (Cruz-Jentoft et al., 2019), the assessment of muscle quality is receiving increasing emphasis. In the Multi-Ethnic Study of Atherosclerosis (MESA), larger abdominal muscle area was associated with more deleterious features of coronary artery calcification (CAC) (larger CAC volume, lower CAC density). This may be because high muscle mass in obese individuals corresponds to more low-density muscle (0–34 HU), and decreased muscle density might increase disease risk (Crawford et al., 2020). Therefore, muscle quantity may not accurately reflect CVD risk. Growing evidence suggests that muscle quality, reflecting muscle composition and intramuscular fat infiltration, is more critical than muscle quantity when considering skeletal muscle function and the risk of adverse outcomes (Crawford et al., 2020; Cruz-Jentoft et al., 2019; Czigany et al., 2021). Lee et al. found that a higher proportion of high-quality muscle was associated with a lower prevalence of severe CAC (Lee et al., 2021). Our previous research also found that in initial dialysis patients, low SMD (low muscle quality) at the L1 level, rather than low SMI (low muscle quantity), was associated with a higher risk of cardiac death (Sheng et al., 2023). From a mechanistic perspective, intramuscular fat infiltration has been linked to insulin resistance, mitochondrial dysfunction, and oxidative stress (Tanaka et al., 2020; Kelley et al., 2002). These pathological processes are well-established drivers of CVD and may underlie the observed association between poor muscle quality and adverse cardiovascular outcomes in dialysis patients.

Several prior studies have reported various CVD mortality prediction models for dialysis patients (Yu et al., 2018; Yang et al., 2023; Li et al., 2022; Anker et al., 2016; Xia et al., 2017; Xu et al., 2024), but the predictive performance of these models still has room for improvement. For example, one model had AUC values of only 0.702, 0.695, and 0.677 for 3-year, 5-year, and 8-year predictions, respectively. Another model had an external validation AUC of 0.73, indicating limited generalizability. Furthermore, existing prediction models are primarily constructed using traditional methods, with most studies only performing internal validation and lacking systematic external dataset validation. The stability and practical application value of these models remain unclear. Therefore, constructing a CVD mortality risk prediction model for dialysis patients with higher predictive performance and validated across multiple external centers holds significant clinical importance. Machine learning can uncover associations between variables and outcomes by learning from high-dimensional clinical data and apply learned patterns to new, unseen data. Moreover, machine learning techniques possess the capability to discover potential unknown patterns, such as identifying novel prognostic markers. This study utilized eight clinical variables to construct machine learning models for predicting CVD mortality risk, and the CatBoost model showed the best predictive performance. CatBoost is a tree-based ensemble approach that performs well in clinical datasets with mixed variable types and marked heterogeneity (Hancock and Khoshgoftaar, 2020). Previous studies have demonstrated strong predictive performance of CatBoost in diverse medical settings (Rong et al., 2025; Cui et al., 2025; Zheng et al., 2023). This model showed strong discrimination and calibration across both internal and external multicenter validation cohorts, and decision curve analysis indicated superior net clinical benefit across most risk thresholds. The final CatBoost model was further combined with SHAP analysis to improve interpretability and facilitate clinical understanding. To facilitate clinical use, we developed a web-based risk calculator with a simple and intuitive interface. The tool enables rapid risk estimation after input of key variables and may assist early identification of patients at high risk of CVD mortality during the initial dialysis period.

This study has certain limitations. Firstly, this was a retrospective study, and the available clinical data were limited. Several relevant variables were not captured, including residual renal function, dialysis prescription, Kt/V, and cardiac biomarkers such as CK-MB, NT-proBNP, and troponin T, which may be informative for CVD mortality risk in initial dialysis patients. Future prospective studies with more complete data collection may help clarify the added value of these indicators and further improve risk prediction. Secondly, this study only included Chinese dialysis patients; whether the findings can be generalized to other ethnicities requires further validation. Thirdly, lifestyle factors such as diet and exercise were not incorporated into the analysis in this study, yet these factors may also play significant roles in the occurrence of CVD mortality.

## Conclusion

In summary, this study identified eight key predictors of CVD-related mortality in initial dialysis patients, encompassing both traditional and nontraditional factors. A machine learning model based on these variables showed good predictive performance, with CatBoost performing best. We also developed a visual online tool for individualized risk assessment. The tool provides risk estimates based on key clinical variables and may support early identification of high-risk patients after dialysis initiation and targeted preventive strategies.

## Data Availability

The raw data supporting the conclusions of this article will be made available by the authors, without undue reservation.
