# Supplementary material for: Developing an explainable machine learning model using body composition to predict cardiovascular mortality in initial dialysis patients: a multicenter study
Source: Front Physiol. 2026 Feb 18;17:1769240. doi: 10.3389/fphys.2026.1769240 (PMC12956525; doi:10.3389/fphys.2026.1769240)
Supplement: Supplementary file 1 [file Supplementaryfile1.docx]

**Table S1 Extent of missing data in the original dataset**

| Variables | Missing values count | Missing values rate |
| --- | --- | --- |
| BMI | 9 | 0.86% |
| WBC | 8 | 0.76% |
| Hemoglobin | 8 | 0.76% |
| PLT | 10 | 0.95% |
| Albumin | 12 | 1.14% |
| FPG | 7 | 0.67% |
| Uric acid | 1 | 0.10% |
| Triglycerides | 45 | 4.28% |
| Total cholesterol | 46 | 4.38% |
| LDL cholesterol | 49 | 4.66% |
| CysC | 224 | 21.31% |
| BUN | 61 | 5.80% |

BMI indicates body mass index; WBC, white blood cell count; Hb, hemoglobin; PLT, platelet count; ALB, albumin; FPG, fasting plasma glucose; UA, uric acid; TG, triglycerides; TC, total cholesterol; LDL-C, low-density lipoprotein cholesterol; CysC, cystatin C; BUN, blood urea nitrogen.

**Table S2 Characteristics of participants in the external validation dataset**

| Characteristic | Total (N=406) | CVD death (N=40) | Non-CVD death (N=366) | *P* value | SMD (Std.)* |
| --- | --- | --- | --- | --- | --- |
| Age, years | 52.0 (42.0–63.0) | 64.0 (52.0–70.5) | 51.0 (41.0–62.0) | <0.001 | −0.815 |
| Sex, n (%) |  |  |  | 0.271 | −0.183 |
| Male | 273 (67.2%) | 30 (75.0%) | 243 (66.4%) |  |  |
| Female | 133 (32.8%) | 10 (25.0%) | 123 (33.6%) |  |  |
| BMI, kg/m^2^ | 22.6 (20.7–24.5) | 23.1 (21.4–25.3) | 22.5 (20.6–24.5) | 0.321 | −0.083 |
| Smoking history, n (%) | 108 (10.3%) | 17 (42.5%) | 91 (24.9%) | 0.017 | −0.401 |
| Alcohol history, n (%) | 46 (4.4%) | 8 (20.0%) | 38 (10.4%) | 0.068 | −0.304 |
| Dialysis methods, n (%) |  |  |  | 0.873 | −0.027 |
| Hemodialysis | 249 (61.3%) | 25 (62.5%) | 224 (61.2%) |  |  |
| Peritoneal dialysis | 157 (38.7%) | 15 (37.5%) | 142 (38.8%) |  |  |
| β-blockers, n (%) | 235 (22.4%) | 16 (40.0%) | 219 (59.8%) | 0.016 | 0.404 |
| ACEI/ARB, n (%) | 39 (3.7%) | 2 (5.0%) | 37 (10.1%) | 0.298 | 0.173 |
| CCB, n (%) | 364 (34.6%) | 34 (85.0%) | 330 (90.2%) | 0.309 | 0.169 |
| Diuretics, n (%) | 184 (17.5%) | 25 (2.5%) | 159 (43.4%) | 0.022 | −0.384 |
| EPO, n (%) | 342 (32.5%) | 35 (87.5%) | 307 (83.9%) | 0.551 | −0.099 |
| Iron agent, n (%) | 115 (10.9%) | 5 (12.5%) | 110 (30.1%) | 0.019 | 0.391 |
| Antiplatelet agents, n (%) | 41 (3.9%) | 8 (20.0%) | 33 (9.0%) | 0.029 | −0.366 |
| Compound α-keto acid, n (%) | 264 (25.1%) | 23 (57.5%) | 241 (65.8%) | 0.293 | 0.175 |
| Glucocorticoids, n (%) | 32 (3.0%) | 2 (5.0%) | 30 (8.2%) | 0.476 | 0.118 |
| Diabetes mellitus, n (%) | 121 (11.5%) | 19 (47.5%) | 102 (27.9%) | 0.010 | −0.432 |
| Hypertension, n (%) | 365 (34.7%) | 35 (87.5%) | 330 (90.2%) | 0.595 | 0.088 |
| Coronary artery disease, n (%) | 27 (2.6%) | 10 (25.0%) | 17 (4.6%) | <0.001 | −0.840 |
| Chronic heart failure, n (%) | 93 (8.8%) | 23 (57.5%) | 70 (19.1%) | <0.001 | −0.947 |
| Stroke, n (%) | 42 (4.0%) | 12 (30.0%) | 30 (8.2%) | <0.001 | −0.731 |
| Cardiac intervention, n (%) | 8 (0.8%) | 1 (2.5%) | 7 (1.9%) | 0.800 | −0.042 |
| CVD, n (%) | 109 (10.4%) | 26 (65.0%) | 83 (22.7%) | <0.001 | −0.994 |
| Hyperlipidemia, n (%) | 72 (6.9%) | 4 (10.0%) | 68 (18.6%) | 0.177 | 0.225 |
| Anemia history, n (%) | 334 (31.8%) | 32 (80.0%) | 302 (82.5%) | 0.693 | 0.066 |
| WBC, *10^9^/L | 6.2 (5.0–7.8) | 5.8 (5.0–7.8) | 6.2 (5.0–7.9) | 0.955 | −0.123 |
| Hemoglobin, g/L | 85.0 (76.0–95.0) | 83.0 (77.5–87.0) | 85.0 (76.0–96.0) | 0.087 | 0.402 |
| PLT, *10^9^/L | 165.5 (127.5–214.3) | 166.5 (147.0–230.0) | 164.5 (126.0–213.0) | 0.207 | −0.163 |
| Albumin, g/L | 32.5 (28.8–35.7) | 30.6 (26.8–34.1) | 32.7 (29.0–35.9) | 0.014 | 0.354 |
| FPG, mg/dl | 5.4 (4.6–6.8) | 6.0 (5.2–7.7) | 5.3 (4.6–6.8) | 0.028 | −0.365 |
| Uric acid, mmol/L | 456.3±143.4 | 459.5±153.6 | 455.9±142.5 | 0.879 | −0.025 |
| Triglycerides, mmol/L | 1.5 (1.0–2.1) | 2.0 (1.1–2.5) | 1.5 (1.0–2.0) | 0.078 | −0.187 |
| Total cholesterol, mmol/L | 4.2 (3.5–4.9) | 4.2 (3.7–5.5) | 4.2 (3.4–4.9) | 0.173 | −0.193 |
| LDL cholesterol, mmol/L | 2.1 (1.7–2.7) | 2.1 (1.9–2.6) | 2.1 (1.6–2.7) | 0.801 | −0.036 |
| SCr, mg/dL | 9.0 (7.5–11.3) | 8.0 (6.7–10.6) | 9.1 (7.6–11.3) | 0.025 | 0.373 |
| CysC, mg/L | 5.3 (4.5–6.3) | 5.2 (3.9–6.9) | 5.3 (4.5–6.3) | 0.610 | 0.081 |
| SCr/CysC | 17.6 (14.0–22.4) | 16.7 (12.7–22.5) | 17.6 (14.2–22.4) | 0.400 | 0.187 |
| BUN, mmol/L | 27.2 (20.1–36.5) | 26.2 (19.6–32.1) | 27.5 (20.1–37.1) | 0.186 | 0.252 |
| SMI, cm^2^/m^2^ | 35.4 (30.4–40.9) | 36.4 (33.0–40.6) | 35.3 (30.4–40.9) | 0.380 | −0.040 |
| SMD, HU | 40.1 (34.0–45.4) | 36.7 (30.8–39.4) | 40.8 (34.4–46.0) | <0.001 | 0.662 |
| LAMA, cm^2^ | 31.9 (24.7–42.9) | 39.2 (30.5–49.1) | 31.3 (24.5–41.4) | 0.005 | −0.425 |
| LAMD, HU | 7.0±2.7 | 6.5±2.5 | 7.0±2.7 | 0.276 | 0.182 |
| LAMA/SMA | 0.3 (0.3–0.4) | 0.4 (0.3–0.5) | 0.3 (0.3–0.4) | 0.002 | −0.544 |
| SFA, cm^2^ | 59.7 (36.6–89.2) | 70.4 (40.6–112.7) | 59.4 (35.8–88.6) | 0.219 | −0.150 |
| SFD, HU | −81.5 (−91.3 – −70.8) | −82.8 (−90.3 – −73.0) | −81.4 (−91.7 – −70.8) | 0.747 | −0.086 |
| VFA, cm^2^ | 58.5 (26.6–111.7) | 82.5 (36.9–129.2) | 57.9 (26.2–109.9) | 0.152 | −0.229 |
| VFD, HU | −83.8 (−90.7 – −76.9) | −83.8 (−91.6 – −74.6) | −83.8 (−90.5 – −76.9) | 0.868 | −0.086 |
| TFA, cm^2^ | 146.1 (84.5–231.5) | 205.3 (91.6–263.1) | 145.1 (84.0–228.8) | 0.256 | −0.134 |
| TFD, HU | −78.6 (−88.0 – −69.5) | −80.5 (−87.9 – −69.1) | −78.5 (−88.0 – −69.5) | 0.916 | −0.075 |

* SMD (Std.): Standardized mean difference (effect size measure). BMI indicates body mass index; ACEI, angiotensin-converting enzyme inhibitor; ARB, angiotensin II receptor blocker; CCB, calcium channel blocker; EPO, erythropoietin; CVD, cardiovascular disease; WBC, white blood cell count; PLT, platelet count; FPG, fasting plasma glucose; LDL, low-density lipoprotein; SCr, serum creatinine; CysC, cystatin C; BUN, blood urea nitrogen; SMI, skeletal muscle index; SMD, skeletal muscle radiodensity; LAMA, low-attenuation muscle area; LAMD, low-attenuation muscle density; SMA, skeletal muscle area; SFA, subcutaneous fat area; SFD, subcutaneous fat density; VFA, visceral fat area; VFD, visceral fat density; TFA, total fat area; TFD, total fat density.

**Table S3 Comparison of patient characteristics between the development dataset and the external validation set**

| Characteristic | Total (N=1051) | The development dataset (N=645) | The external validation set (N=406) | *P* value | SMD (Std.)* |
| --- | --- | --- | --- | --- | --- |
| Age, years | 54 (44–64) | 55 (45–65) | 52 (42–63) | 0.006 | −0.161 |
| Sex, n (%) |  |  |  | 0.049 | 0.125 |
| Male | 668 (63.6%) | 395 (61.2%) | 273 (67.2%) |  |  |
| Female | 383 (36.4%) | 250 (38.8%) | 133 (32.8%) |  |  |
| BMI, kg/m^2^ | 23.3 (21.0–25.9) | 23.8 (21.3–26.5) | 22.6 (20.7–24.5) | <0.001 | −0.296 |
| Smoking history, n (%) | 223 (21.2%) | 115 (17.8%) | 108 (26.6%) | <0.001 | 0.216 |
| Alcohol history, n (%) | 94 (8.9%) | 48 (7.4%) | 46 (11.3%) | 0.032 | 0.136 |
| Dialysis methods, n (%) |  |  |  | <0.001 | −0.693 |
| Hemodialysis | 820 (78.0%) | 571 (88.5%) | 249 (61.3%) |  |  |
| Peritoneal dialysis | 231 (22.0%) | 74 (11.5%) | 157 (38.7%) |  |  |
| β-blockers, n (%) | 633 (60.2%) | 398 (61.7%) | 235 (57.9%) | 0.218 | −0.078 |
| ACEI/ARB, n (%) | 257 (24.5%) | 218 (33.8%) | 39 (9.6%) | <0.001 | −0.585 |
| CCB, n (%) | 920 (87.5%) | 556 (86.2%) | 364 (89.7%) | 0.099 | 0.105 |
| Diuretics, n (%) | 446 (42.4%) | 262 (40.6%) | 184 (45.3%) | 0.133 | 0.095 |
| EPO, n (%) | 891 (84.8%) | 549 (85.1%) | 342 (84.2%) | 0.699 | −0.024 |
| Iron agent, n (%) | 483 (46.0%) | 368 (57.1%) | 115 (28.3%) | <0.001 | −0.600 |
| Antiplatelet agents, n (%) | 208 (19.8%) | 167 (25.9%) | 41 (10.1%) | <0.001 | −0.404 |
| Compound α-keto acid, n (%) | 537 (51.1%) | 273 (42.3%) | 264 (65.0%) | <0.001 | 0.465 |
| Glucocorticoids, n (%) | 123 (11.7%) | 91 (14.1%) | 32 (7.9%) | 0.002 | −0.194 |
| Diabetes mellitus, n (%) | 419 (39.9%) | 298 (46.2%) | 121 (29.8%) | <0.001 | −0.339 |
| Hypertension, n (%) | 955 (90.9%) | 590 (91.5%) | 365 (89.9%) | 0.389 | −0.055 |
| Coronary artery disease, n (%) | 152 (14.5%) | 125 (19.4%) | 27 (6.7%) | <0.001 | −0.367 |
| Chronic heart failure, n (%) | 290 (27.6%) | 197 (30.5%) | 93 (22.9%) | 0.007 | −0.171 |
| Stroke, n (%) | 105 (10.0%) | 63 (9.8%) | 42 (10.3%) | 0.761 | 0.019 |
| Cardiac intervention, n (%) | 46 (4.4%) | 38 (5.9%) | 8 (2.0%) | 0.002 | −0.192 |
| CVD, n (%) | 406 (38.6%) | 297 (46.0%) | 109 (26.8%) | <0.001 | −0.401 |
| Hyperlipidemia, n (%) | 109 (10.4%) | 37 (5.7%) | 72 (17.7%) | <0.001 | 0.401 |
| Anemia history, n (%) | 876 (83.3%) | 542 (84.0%) | 334 (82.3%) | 0.455 | −0.047 |
| WBC, *10^9^/L | 6.4 (5.2–8.2) | 6.6 (5.3–8.3) | 6.2 (5.0–7.8) | 0.011 | −0.163 |
| Hemoglobin, g/L | 84.0 (75.0–95.0) | 83.0 (74.0–94.0) | 85.0 (76.0–95.0) | 0.069 | 0.036 |
| PLT, *10^9^/L | 167.0 (125.0–213.0) | 169.0 (124.0–209.0) | 165.5 (128.0–214.0) | 0.832 | 0.008 |
| Albumin, g/L | 32.9 (29.2–36.5) | 33.2 (29.6–37.1) | 32.5 (28.8–35.7) | 0.002 | −0.192 |
| FPG, mg/dl | 5.4 (4.6–6.8) | 5.4 (4.6–6.9) | 5.4 (4.6–6.8) | 0.828 | −0.057 |
| Uric acid, mmol/L | 462.2 (368.7–554) | 466.0 (378.7–561.0) | 454.8 (352.5–540.3) | 0.027 | −0.153 |
| Triglycerides, mmol/L | 1.5 (1.1–2.0) | 1.4 (1.1–2.0) | 1.5 (1.0–2.1) | 0.524 | 0.030 |
| Total cholesterol, mmol/L | 4.1 (3.3–4.9) | 4.0 (3.3–4.9) | 4.2 (3.5–4.9) | 0.151 | 0.040 |
| LDL cholesterol, mmol/L | 2.2 (1.8–2.9) | 2.3 (1.8–3.0) | 2.1 (1.7–2.7) | 0.001 | −0.216 |
| SCr, mg/dL | 9.0 (7.6–11.4) | 9.0 (7.6–11.5) | 9.0 (7.5–11.3) | 0.418 | −0.117 |
| CysC, mg/L | 5.1 (4.1–6.2) | 4.9 (3.8–6.2) | 5.3 (4.5–6.3) | <0.001 | 0.199 |
| SCr/CysC | 18.8 (14.2–23.9) | 19.6 (14.5–25.1) | 17.6 (14.0–22.4) | <0.001 | −0.308 |
| BUN, mmol/L | 28.3 (20.7–36.5) | 28.8 (21.4–36.3) | 27.2 (20.1–36.5) | 0.217 | −0.099 |
| SMI, cm^2^/m^2^ | 38.5 (32.3–44.2) | 40.3 (34.6–45.2) | 35.4 (30.4–40.9) | <0.001 | −0.494 |
| SMD, HU | 36.2±8.8 | 34.1±8.3 | 39.6±8.4 | <0.001 | 0.663 |
| LAMA, cm^2^ | 40.5 (30.2–54.9) | 46.6 (36.6–59.3) | 31.9 (24.8–42.7) | <0.001 | −0.822 |
| LAMD, HU | 6.6 (4.8–8.3) | 6.4 (4.6–7.9) | 7.0 (5.3–8.7) | <0.001 | 0.295 |
| LAMA/SMA | 0.4 (0.3–0.5) | 0.4 (0.4–0.5) | 0.3 (0.3–0.4) | <0.001 | −0.683 |
| SFA, cm^2^ | 66.2 (40.3–104.6) | 70.4 (43.8–110.6) | 59.7 (36.6–89.1) | <0.001 | −0.252 |
| SFD, HU | −82.9 (−93.0 – −72.1) | −84.0 (−94.5 – −73.4) | −81.5 (−91.2 – −70.8) | 0.009 | 0.181 |
| VFA, cm^2^ | 69.5 (31.2–126.9) | 75.0 (33.9–135.2) | 58.5 (26.7–111.7) | 0.001 | −0.198 |
| VFD, HU | −84.6 (−92.1 – −77.1) | −85.5 (−93.0 – −77.5) | −83.8 (−90.7 – −76.9) | 0.007 | 0.187 |
| TFA, cm^2^ | 171.3 (97.3–265.2) | 185.9 (105.8–284.1) | 146.1 (84.7–231.3) | <0.001 | −0.301 |
| TFD, HU | −79.7 (−89.0 – −69.4) | −80.8 (−89.9 – −69.4) | −78.6 (−88.0 – −69.5) | 0.038 | 0.134 |

* SMD (Std.): Standardized mean difference (effect size measure). BMI indicates body mass index; ACEI, angiotensin-converting enzyme inhibitor; ARB, angiotensin II receptor blocker; CCB, calcium channel blocker; EPO, erythropoietin; CVD, cardiovascular disease; WBC, white blood cell count; PLT, platelet count; FPG, fasting plasma glucose; LDL, low-density lipoprotein; SCr, serum creatinine; CysC, cystatin C; BUN, blood urea nitrogen; SMI, skeletal muscle index; SMD, skeletal muscle radiodensity; LAMA, low-attenuation muscle area; LAMD, low-attenuation muscle density; SMA, skeletal muscle area; SFA, subcutaneous fat area; SFD, subcutaneous fat density; VFA, visceral fat area; VFD, visceral fat density; TFA, total fat area; TFD, total fat density.

**Table S4 Collinearity diagnosis: tolerance and variance inflation factor (VIF)**

| Variables | Collinearity statistics | |
| --- | --- | --- |
|  | Tolerance | VIF |
| Age, years | 0.680 | 1.471 |
| Hemoglobin, g/L | 0.990 | 1.010 |
| SCr, mg/dL | 0.838 | 1.194 |
| SMD, HU | 0.750 | 1.334 |
| Diabetes mellitus | 0.855 | 1.170 |
| CVD | 0.806 | 1.241 |
| Cardiac intervention | 0.912 | 1.096 |
| Dialysis modality | 0.930 | 1.075 |

SCr indicates serum creatinine; SMD, skeletal muscle density; CVD, cardiovascular disease.


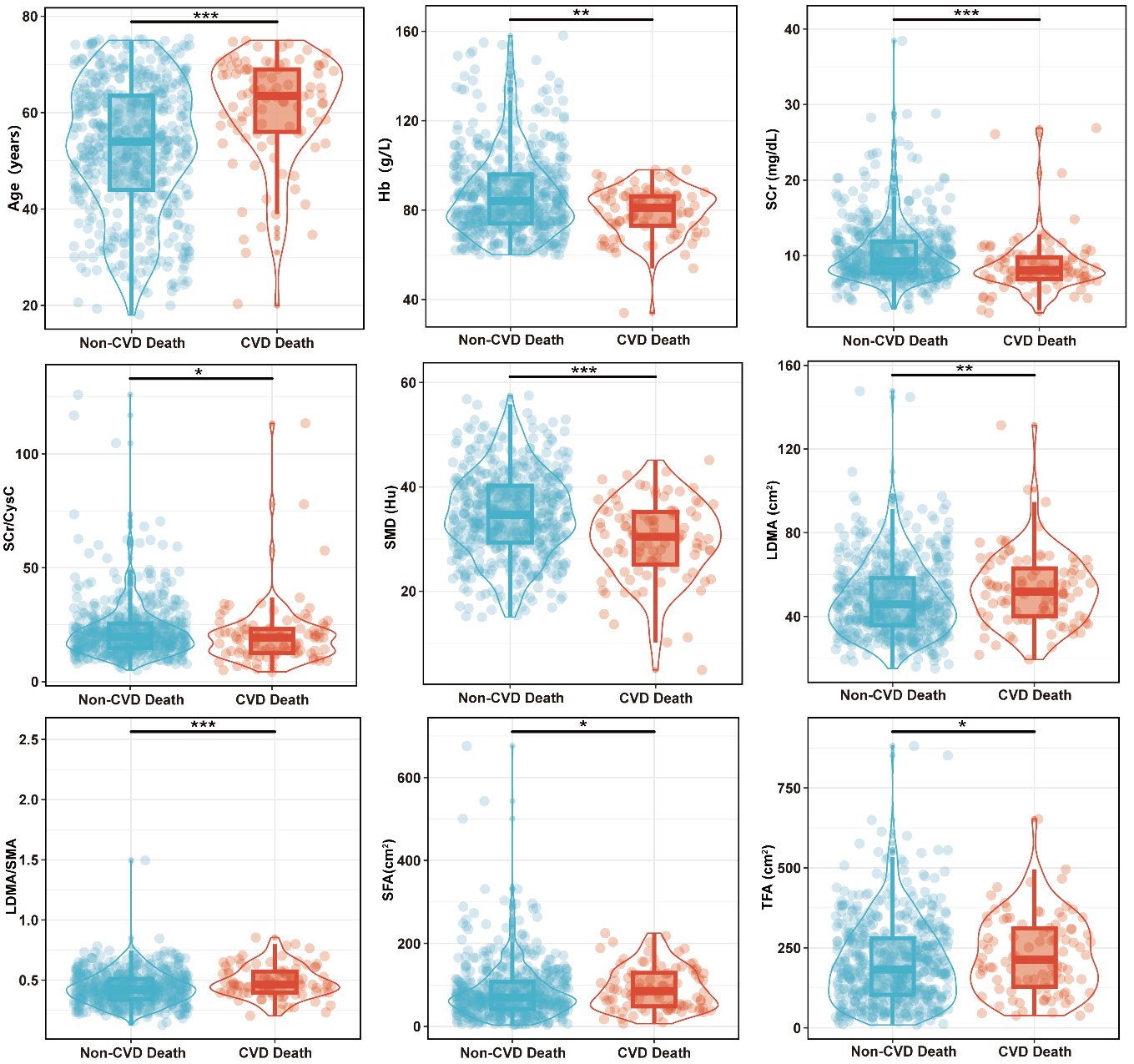


**Figure S1 Violin plots showing the distribution of differential variables between the CVD death group and the non-CVD death group**


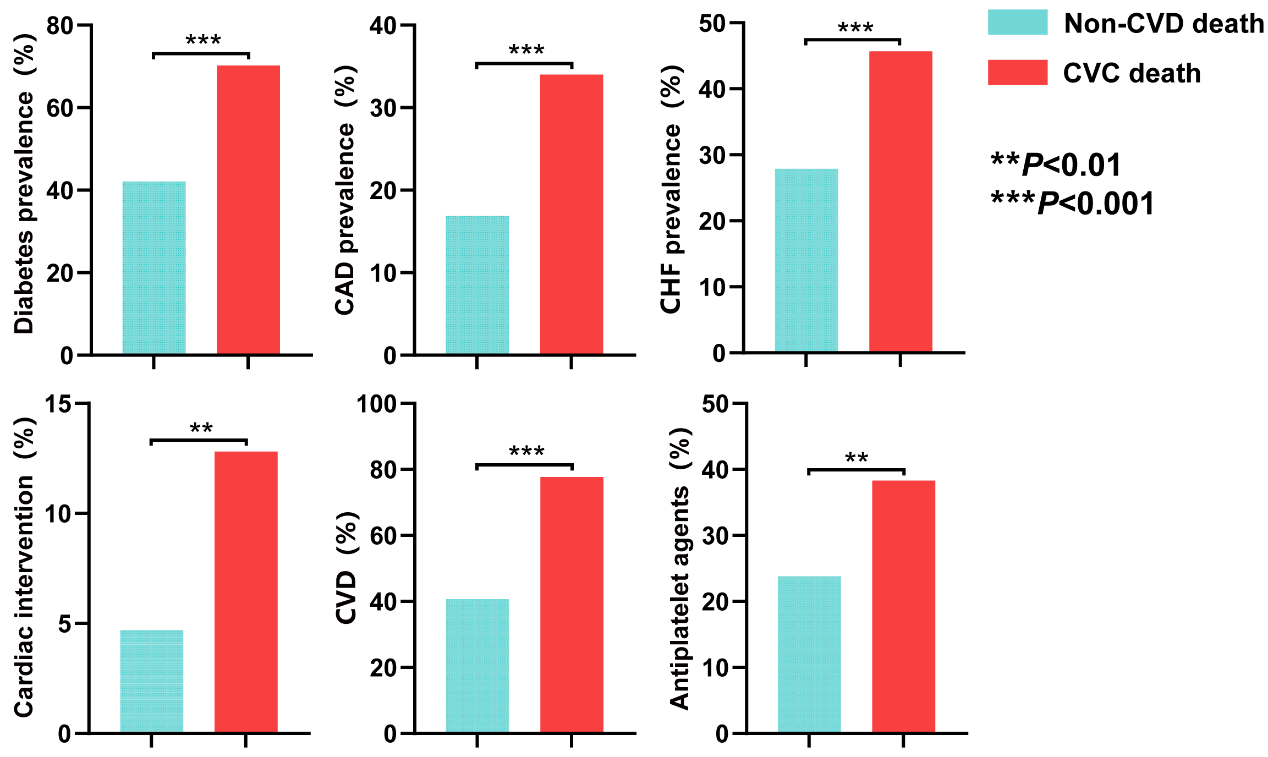


**Figure S2 Comparison of comorbidities and medication use between the CVD death and non-CVD death groups**


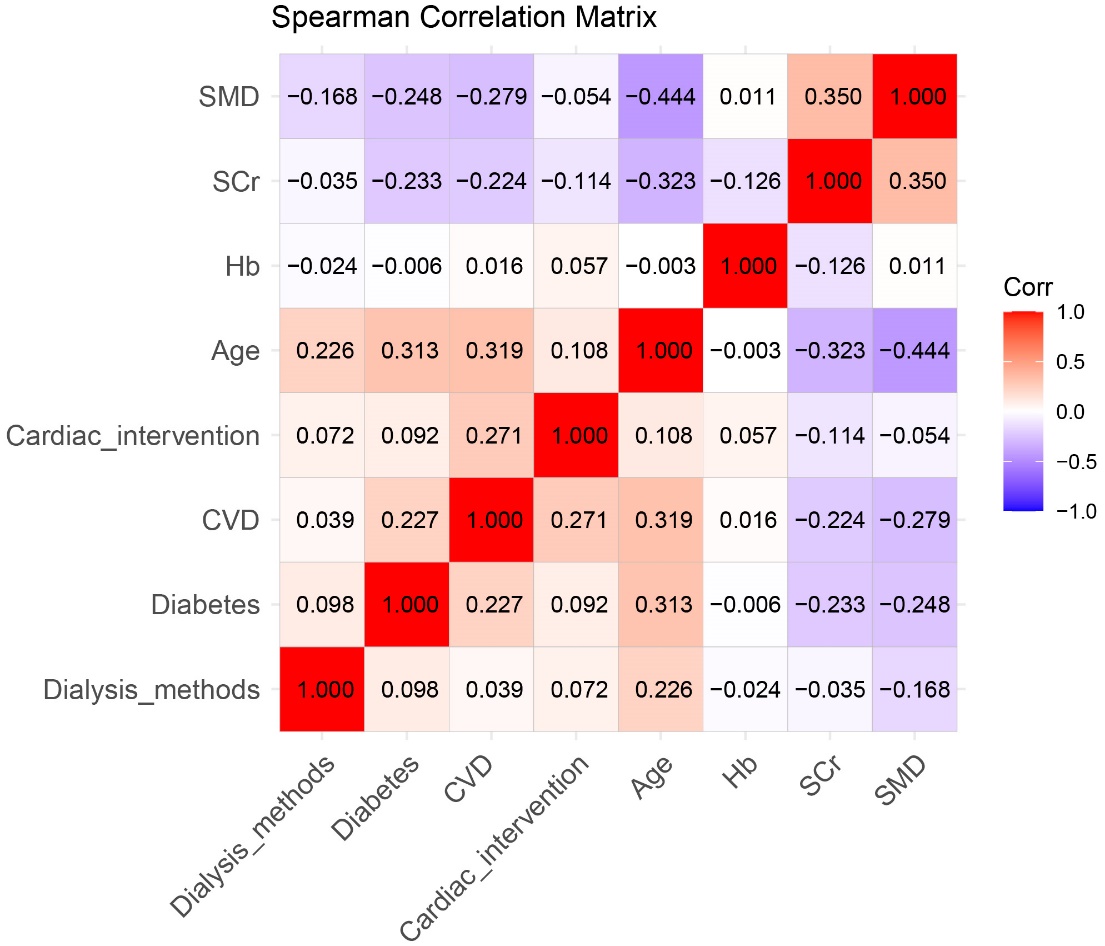


**Figure S3 Heatmap of feature correlations**


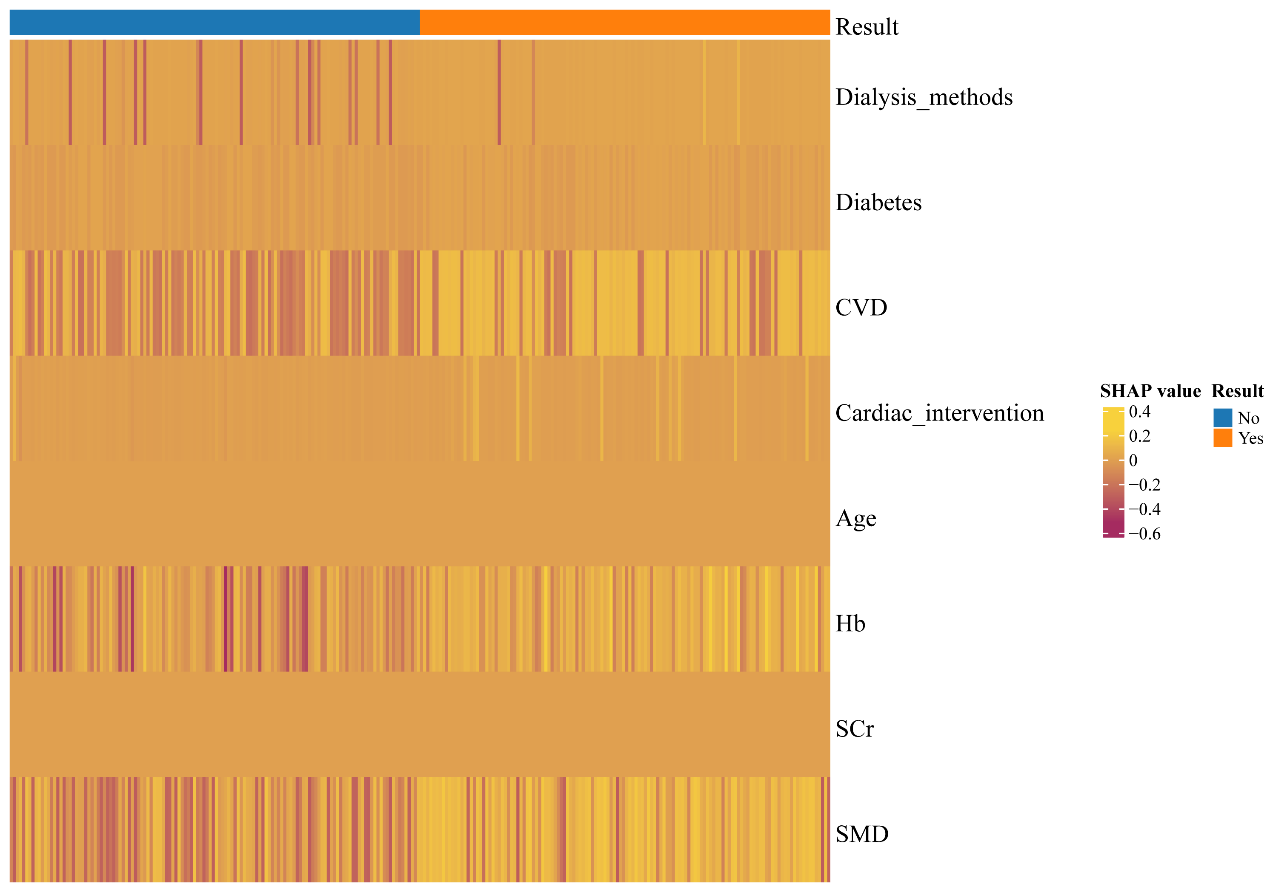


**Figure S4 SHAP value heatmap showing individual-level feature contributions ordered by predicted outcome**


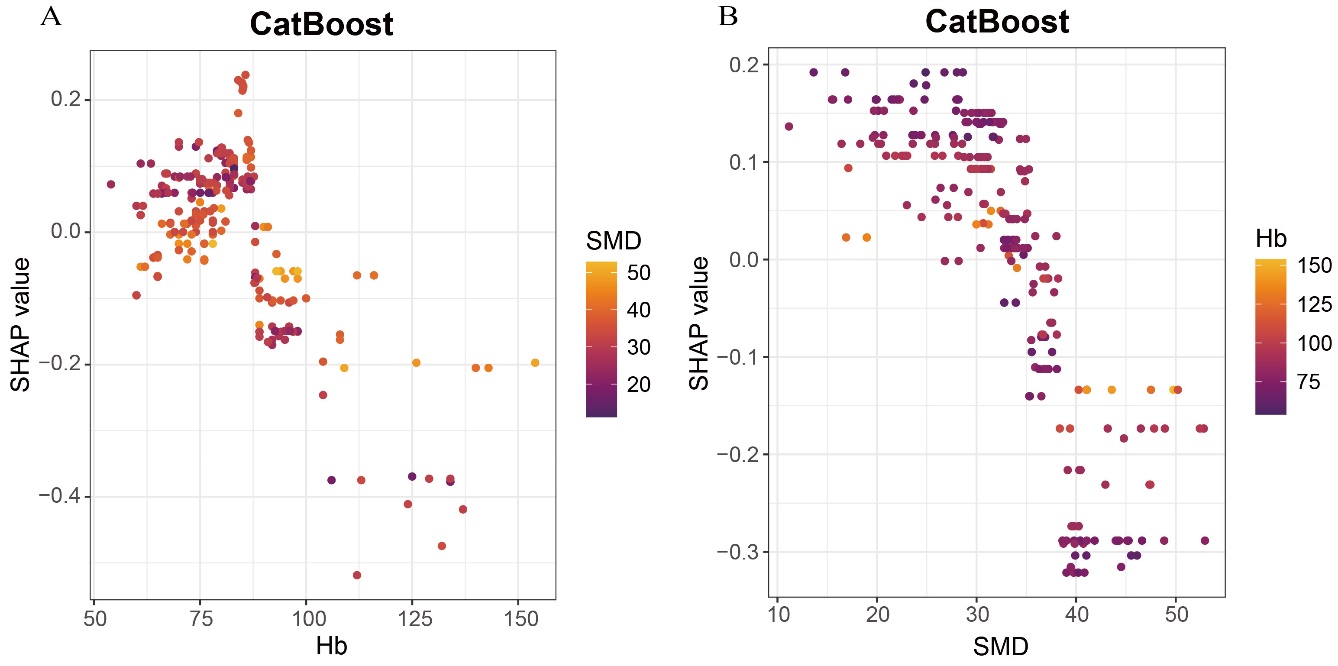


**Figure S5 SHAP interaction analysis showing the combined influence of Hb and SMD on CatBoost model predictions**
